# RNA Viruses Linked to Eukaryotic Hosts in Thawed Permafrost

**DOI:** 10.1128/msystems.00582-22

**Published:** 2022-12-01

**Authors:** Ruonan Wu, Eric M. Bottos, Vincent G. Danna, James C. Stegen, Janet K. Jansson, Michelle R. Davison

**Affiliations:** a Earth and Biological Sciences Directorate, Pacific Northwest National Lab, Richland, Washington, USA; b Department of Biological Sciences, Thompson Rivers University, Kamloops, British Columbia, Canada; Institute of soil science, Chinese academy of sciences

**Keywords:** RNA virus, metatranscriptomics, permafrost thaw, soil virus

## Abstract

Arctic permafrost is thawing due to global warming, with unknown consequences on the microbial inhabitants or associated viruses. DNA viruses have previously been shown to be abundant and active in thawing permafrost, but little is known about RNA viruses in these systems. To address this knowledge gap, we assessed the composition of RNA viruses in thawed permafrost samples that were incubated for 97 days at 4°C to simulate thaw conditions. A diverse RNA viral community was assembled from metatranscriptome data including double-stranded RNA viruses, dominated by *Reoviridae* and *Hypoviridae*, and negative and positive single-stranded RNA viruses, with relatively high representations of *Rhabdoviridae* and *Leviviridae*, respectively. Sequences corresponding to potential plant and human pathogens were also detected. The detected RNA viruses primarily targeted dominant eukaryotic taxa in the samples (e.g., fungi, *Metazoa* and *Viridiplantae*) and the viral community structures were significantly associated with predicted host populations. These results indicate that RNA viruses are linked to eukaryotic host dynamics. Several of the RNA viral sequences contained auxiliary metabolic genes encoding proteins involved in carbon utilization (e.g., polygalacturosase), implying their potential roles in carbon cycling in thawed permafrost.

**IMPORTANCE** Permafrost is thawing at a rapid pace in the Arctic with largely unknown consequences on ecological processes that are fundamental to Arctic ecosystems. This is the first study to determine the composition of RNA viruses in thawed permafrost. Other recent studies have characterized DNA viruses in thawing permafrost, but the majority of DNA viruses are bacteriophages that target bacterial hosts. By contrast RNA viruses primarily target eukaryotic hosts and thus represent potential pathogenic threats to humans, animals, and plants. Here, we find that RNA viruses in permafrost are novel and distinct from those in other habitats studied to date. The COVID-19 pandemic has heightened awareness of the importance of potential environmental reservoirs of emerging RNA viral pathogens. We demonstrate that some potential pathogens were detected after an experimental thawing regime. These results are important for understanding critical viral-host interactions and provide a better understanding of the ecological roles that RNA viruses play as permafrost thaws.

## INTRODUCTION

Permafrost contains diverse microbial life, including bacteria, archaea, fungi, protists, and viruses, that are trapped in a frozen state of dormancy or low metabolic activity (1, 2). Increased global temperatures due to climate change have begun to thaw permafrost, reviving microbial populations and allowing sequestered resources, such as carbon, to become accessible for microbial use (3, 4). This results in increased production of greenhouse gases (CO_2_, CH_4_ and N_2_O) in thawed permafrost due to increased microbial metabolism of soil organic matter (1, 2). Several studies have recently conducted investigations of permafrost microbial communities before and after thaw (4–6). Much of this research has leveraged high throughput DNA sequencing technologies and new increasingly powerful bioinformatic workflows (7–9). As a result, these studies are unveiling the previously hidden composition of microbial community members in permafrost (2, 5, 6, 10). Most studies to date have characterized prokaryotic community compositions (5), but some have also identified DNA viruses (6, 10). These studies have revealed a diversity of microorganisms and associated DNA viruses in permafrost before and after thaw.

There is increasing concern that permafrost could be a reservoir of plant and animal pathogens (11). This threat was highlighted by an outbreak of anthrax in humans from infected reindeer carcasses that were exposed during permafrost thaw in Siberia, resulting in several deaths (12, 13). In the case of anthrax, the causative agents are bacteria. However, viable and infective giant DNA viruses from 30000-year-old permafrost in Siberia have also been recovered (14). In this case, the host was a protozoan and did not pose a threat to humans. In fact, the majority of the DNA viruses identified from Arctic soils to date are bacteriophages that target bacterial hosts and thus do not pose a direct human health risk (6, 10, 15). For example, bacteriophages have been detected in metagenomes that were constructed from extracted viral particles (viromes) along a permafrost thaw gradient (6, 10). In addition, active bacteriophages were identified in Arctic thermokarst bog samples by stable isotope probing (SIP)-labeled metagenomics (15). Several DNA viruses incorporated ^18^O into their DNA during incubations with ^18^O-labeled H_2_O during simulated winter conditions at −1.5°C for 370 days (15), suggesting that they were active under those conditions. By contrast, little is currently known about RNA viruses in permafrost. This knowledge gap is disconcerting considering that RNA viruses primarily target eukaryotic hosts and several RNA viruses, such as COVID-19, are human pathogens (16, 17). Two studies to date have screened for soil RNA viruses in RNA sequence data (metatranscriptomes) derived from bulk soils: one from a California grassland (18) and one from a Kansas grassland (19). Another recent study enriched the virome from a range of soil types prior to metatranscriptome sequencing, and revealed a high diversity of soil RNA viruses, including those that infect bacteria, plants, fungi, vertebrates, and invertebrates (20). The enormous diversity of RNA viruses in permafrost and other soil types, however, remains largely unexplored.

Characteristics unique to permafrost thaw may also impact the “arms-race” between viruses and hosts in these systems. Viruses currently entrapped in permafrost may resurface and interact with current host populations, impacting the ecology of the system. For example, viral predation can lead to lysis of susceptible host populations, resulting in compositional shifts of microbial communities (21). In response, host populations can evolve various defense mechanisms, such as clustered regularly interspaced short palindromic repeats (CRISPR)-CRISPR-associated protein (Cas) systems in prokaryotes and antibody receptor diversification in eukaryotes (22). In contrast to DNA viruses, RNA viruses, with quasi-species population dynamics resulting from high mutation rates, and recombination and reassortment frequencies (23), are potentially more responsive to environmental changes. RNA viruses are also known to infect several Eukarya, including fungi, *Viridiplantae*, *Rhizaria*, and *Metazoa*, that have been detected in permafrost sequence data (24). However, the response of RNA viruses to permafrost thaw remains unknown.

Here, we addressed these knowledge gaps by re-constructing RNA viral sequences from metatranscriptomes that were derived from thawed permafrost soil samples. Permafrost soil cores were collected across a boreal forest landscape in central Alaska as previously described (25) and incubated at 4°C for 97 days to represent ~3 month seasonal transition of permafrost to an active layer. This work contributes new insights into how RNA viruses emerge as permafrost thaws and to a growing understanding of ecological controls on microbial distributions in permafrost ecosystems.

## RESULTS AND DISCUSSION

### RNA viruses in thawed permafrost.

RNA viral sequences were recovered from permafrost soil samples after thaw and 97 days of incubation at 4°C. Following incubation, total RNA was harvested and sequenced to generate metatranscriptomes (Table S1). The metatranscriptomes were *de novo* assembled and screened for RNA viral sequences. A total of 22,408 RNA ‘viral-like’ contigs were identified (Table S2). The majority of the contigs (22,171; 98.9%) were annotated by taxonomic assignment to RNA-dependent RNA polymerase (RdRp), a phylogenetic marker used for RNA virus classification (26). The 22,171 RdRp genes were dereplicated into 230 unique RdRp sequences (clustered at 99% amino acid identity) (Table S2). Of the unique RdRp gene sequences, 184 had taxonomic annotations. The RNA viral contigs inherited the taxonomic assignments of the detected or assigned RdRP, which resulted in assignment of 99.8% of the 22,171 RNA viral contigs (22, 124) to 39 families according to the standard 15 hierarchical ranks (27). Unclassified RNA viral contigs only accounted for 2% of the total genome coverage of RNA viruses identified and these were discarded from further analyses. These data contribute valuable new information about the composition of RNA viruses in environmental habitats.

10.1128/msystems.00582-22.1 TABLE S1 Sequencing data statistics and physicochemical properties of permafrost samples. Sequencing statistics, including number of qualified bases and reads, number of assemblies, N50, and number of RNA viral contigs detected from each of the 33 thawed permafrost samples and the measured physicochemical properties of each original frozen permafrost sample. Download Table S1, XLSX file, 0.02 MB. https://doi.org/10.1128/AuthorWarrantyLicense.v1 This is a work of the U.S. Government and is not subject to copyright protection in the United States. Foreign copyrights may apply.

10.1128/msystems.00582-22.2 TABLE S2 Taxonomic assignments and abundance estimates of detected RNA viral contigs. Details of methods applied, and annotation statistics used to assign taxonomy to each detected RNA viral contig, together with their estimated abundances. Download Table S2, XLSX file, 2.2 MB. https://doi.org/10.1128/AuthorWarrantyLicense.v1 This is a work of the U.S. Government and is not subject to copyright protection in the United States. Foreign copyrights may apply.

**FIG 1:**
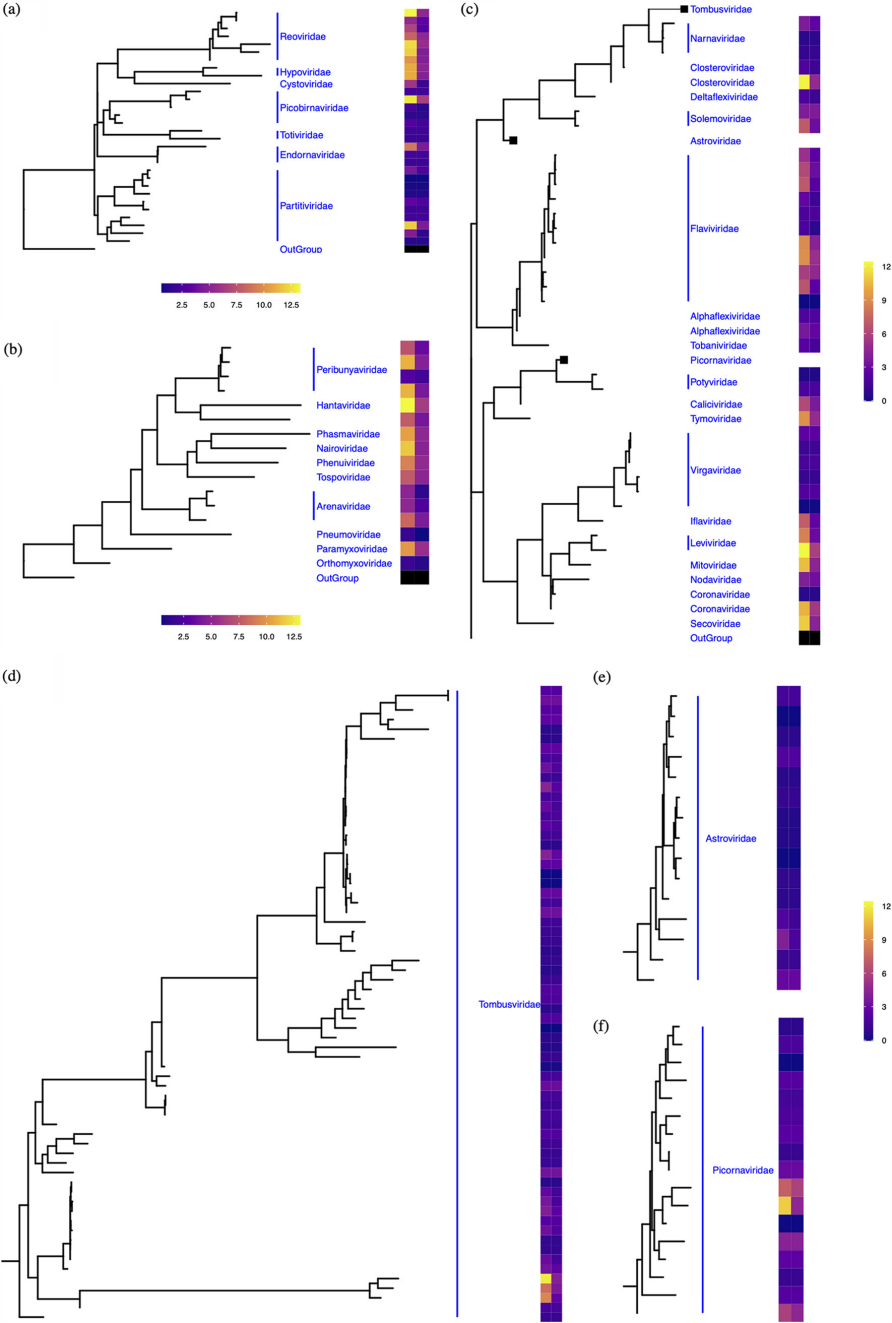
Phylogenetic composition and abundance estimates of RNA viruses detected in thawed permafrost. Phylogenetic trees of the detected double-stranded RNA viruses (a), negative single-stranded RNA viruses (b), and positive single-stranded RNA viruses (c) were reconstructed based on multiple sequence alignments of RNA-dependent RNA polymerase (RdRP) protein sequences and rooted by an RNA directed DNA polymerase (RdDP, APO57079.1) of an Alphaproteobacterium (‘Outgroup’). Due to the high sequence diversity, clades of *Tombusviridae*, *Astroviridae* and *Picornaviridae* were collapsed in panel (c) and displayed in separate panels (d), (e), and (f), respectively. The sum (left column) and average (right column) of the calculated average base-coverage of RNA viral contigs per taxon were used to estimate the relative abundances of the detected RNA viruses.

Three rooted protein trees were derived to determine the phylogenetic placements of the 184 unique RdRp genes (Fig. 1), including double-stranded (ds) RNA viruses (Fig. 1a), negative single-stranded (-ss) RNA viruses (Fig. 1b), and positive single-stranded (+ss) RNA viruses (Fig. 1c). As some RNA viruses are known to have segmented genomes (e.g., *Reoviridae* [28] and *Cystoviridae* [29]), and the genomes assembled from metatranscriptomes are often incomplete, it is challenging to apply the common practice of using total average read coverage to estimate relative abundances of the detected RNA viruses (30). Therefore, we calculated both the sum and average read coverages of RNA viral contigs that were assigned to each Family cluster and aligned them to the respective RdRP sequences of the cluster shown in the tree (Fig. 1, first and second columns of the heatmaps) to represent the upper and lower bounds of the abundance estimates.

The identified dsRNA viruses were dominated by viral contigs annotated as *Reoviridae*, *Hypoviridae*, and members of *Picobirnaviridae* and *Partitiviridae* families (Fig. 1a). By contrast, the (-) ssRNA viruses, which represented 11 families, only showed high abundances for families with few unique members (e.g., *Rhabdoviridae* and *Nairoviridae*). *Rhabdoviridae* was the most abundant (-) ssRNA viral family detected (Fig. 1b). All of the *Rhabdoviridae*-like viral contigs displayed hits to one reference RNA virus, *Guampa vesiculovirus* (MN225577.1) (Table S2). *Guampa vesiculovirus* was previously found in mosquitoes from the Brazilian Pantanal that are known to be critical vectors for transmitting animal diseases (31). Given that *Rhabdoviridae* is one of the most diverse RNA viral families characterized in other habitats (32), the finding of only 1 type of *Rhabdoviridae* in our samples suggests that the detected member could be relatively more adapted to permafrost thaw conditions. The remaining 21 families belonged to (+) ssRNA viruses that had the highest representation of members of *Leviviridae*, *Closteroviridae*, and *Picornaviridae* (Fig. 1c to f). *Tombusviridae* was the most diverse family of RNA viruses detected (Fig. 1d). During the 97-day incubation, the detected RNA viruses were likely enriched along with their respective hosts or preserved within the vectors. The detection of RNA viruses across samples from multiple field transects, therefore, implies their prevalence and high potential for emergence in thawed permafrost. During the extended thaw incubation, RNA turnover could occur. Therefore, the RNA viruses detected after the 97-day incubation represent viruses that were not degraded, but instead were stable or otherwise enriched together with their hosts. Although out of scope of the present study, a future direction would be to compare the RNA viral community in thawed permafrost to that in intact permafrost. This comparison would be useful for determining whether the RNA viruses that we detected were present in the permafrost both pre-and post-thaw.

### RNA viruses in thawed permafrost soils are unique.

**FIG 2:**
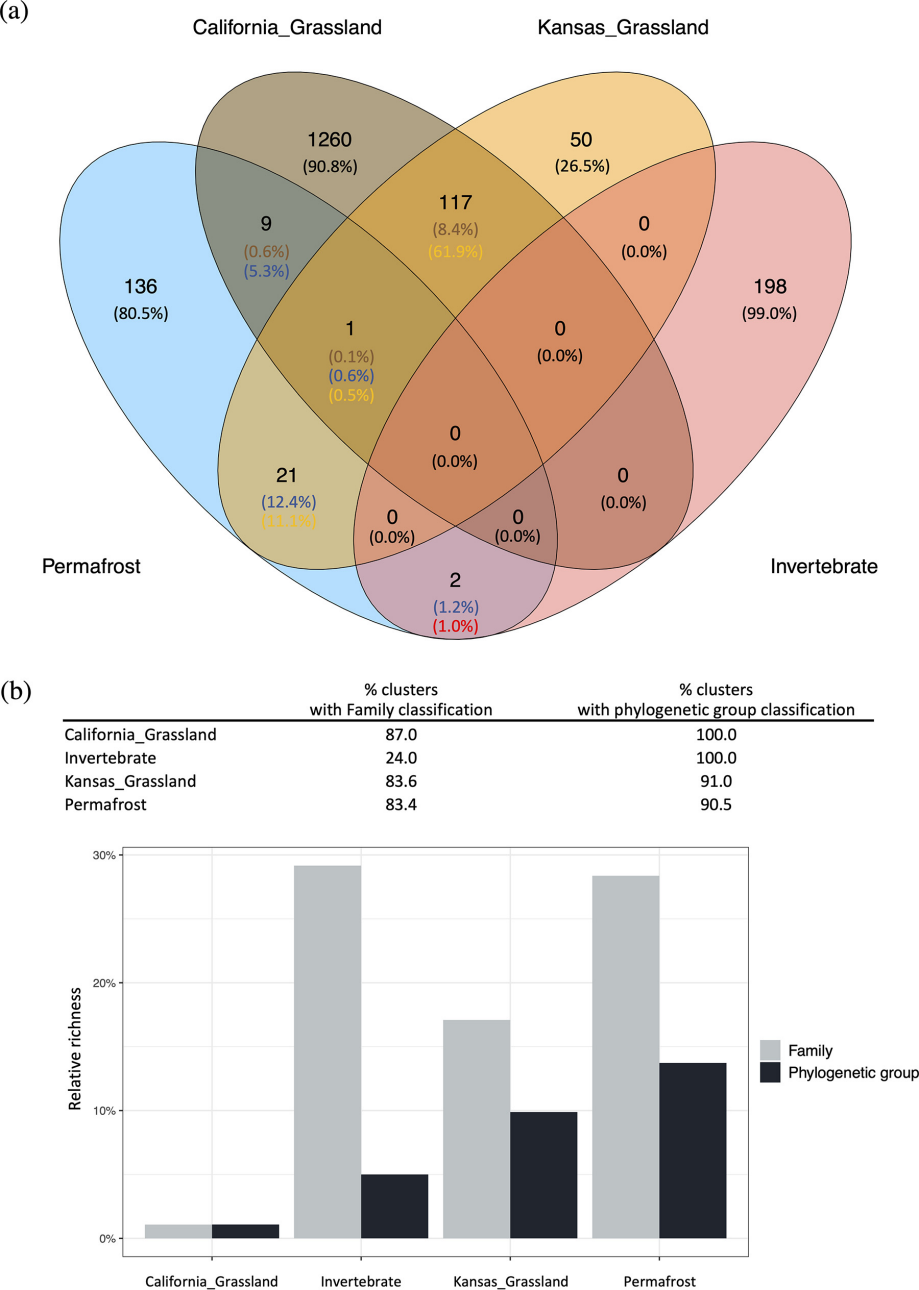
Comparison of RNA viruses across ecosystems. (a) The number of RdRP clusters that were shared and/or unique to each ecosystem are labeled in the respective sections of the Venn diagram. The percentage of shared clusters compared to the total number of clusters for each ecosystem were calculated (blue, thawed permafrost; brown, California grassland; yellow, Kansas grassland; red, invertebrate). The percentage of the clusters that were specific to each ecosystem are labeled in black. (b) The percentage of RdRP clusters with family or phylogenetic group classifications for each ecosystem. Relative richness was calculated at family (gray) and phylogenetic (black) levels.

RNA viruses in terrestrial environments are currently undersampled and understudied with only a few RNA viral studies published to date from terrestrial environments. One study is a comprehensive survey of invertebrate-associated RNA viruses sampled from both terrestrial and freshwater arthropods (Phylum *Arthopoda*) (26) (Fig. 2, ‘Invertebrate’). In addition, there are two grassland soil RNA viral studies to date, one in microcosms constructed from a California grassland soil (18) (Fig. 2, ‘California_Grassland’) and the other from a Kansas native prairie, grassland soil (19) (Fig. 2, ‘Kansas_Grassland’).

To examine whether RNA viruses in thawed permafrost (Fig. 2, ‘Permafrost’) were found in the other terrestrial environments studied to date, a comparison of the detected RNA viruses from the four studies was made. RdRp protein sequences were retrieved from ‘Permafrost’, ‘California_Grassland’, ‘Kansas_Grassland’ and ‘Invertebrate’ data sets, respectively, and de-replicated into unique sequences (at 99% amino acid identity) ranging from 215 to 2191 per data set. We adopted the ‘phylogenetic group’ classification scheme based on published examples (26) to allow cross-study comparisons. A phylogenetic group can contain one or multiple viral families that are phylogenetically relevant. We acknowledge that differences in methodologies applied to each study could contribute to the differences in the absolute counts of detected RNA viruses. To account for this possibility, we compared the percentages of detected RNA viruses that were shared across the four ecosystems. The de-replicated RdRp protein sequences were pooled and grouped into 1795 clusters with a cut-off of 70% amino acid identity to gain insight into RNA virus similarity across ecosystems. We also confirmed that different RNA viral families or phylogenetic groups were not grouping into the same cluster after applying the cut-off. Thus, 70% amino acid identity for clustering RdRp sequences was sufficient to differentiate the detected RNA viruses at the taxonomic level of Family (27) and the categorized phylogenetic group (18, 26).

RNA viruses were distinct per study, with only one cluster detected in more than two ecosystems based on the phylogenetic marker, RdRp. The non-singleton clusters were visualized in a network in Fig. S1, demonstrating the size of these clusters. The network highlights the unique assemblage of RNA viruses in thawed permafrost, with one dominant cluster grouping the majority of the RdRp sequences (40.04% of the non-singleton clustered sequences). The RdRp sequences were detected in 169 clusters with most of the clusters being unique (80.5%) to the thawed permafrost samples. More of the RdRp sequences that were detected in the thawed permafrost were shared with the other soil types, whereas fewer were shared with invertebrate-associated RNA viruses (Fig. 2a). This finding could be expected considering that the invertebrate samples represent a very different habitat type compared to soil. The invertebrate-associated RNA viral RdRp sequences grouped into 200 clusters (Fig. 2a), with few clusters shared with soil samples.

10.1128/msystems.00582-22.4 FIG S1 Clustering network of RNA viruses detected from thawed permafrost, grassland soils and invertebrates. RNA viral contigs detected from thawed permafrost (nodes in blue), California grassland (nodes in brown), Kansas grassland (nodes in yellow) and invertebrates (nodes in red) were clustered and non-singleton clusters are visualized in the network. Download FIG S1, PDF file, 0.2 MB. https://doi.org/10.1128/AuthorWarrantyLicense.v1 This is a work of the U.S. Government and is not subject to copyright protection in the United States. Foreign copyrights may apply.

The California and Kansas grassland RdRp sequences grouped into 1387 and 189 clusters and shared the most RNA viral clusters with each other (8.5% to ‘California_Grassland’ and 62.4% to ‘Kansas_Grassland’) (Fig. 2a). The majority of these shared clusters (117 out of the 118 clusters) were only detected in these two studies, implying the presence of grassland soil-specific RNA viral clusters. However, a significant amount of the grassland RNA viral clusters were unique to each site (90.8% to ‘California_Grassland’ and 26.5% to ‘Kansas_Grassland’) (Fig. 2a), which can be due to the differences in geographic, climate and/or soil environmental conditions. For example, soil moisture has been reported to influence grassland RNA viral community compositions and these two sites have very different historical precipitation regimes, with California representing a drier ‘Mediterranean’ climate when compared to Kansas (33).

The RdRp clusters (‘Viral cluster’) detected from the four biomes (‘Ecosystem’) were then mapped to the assigned phylogenetic groups (‘Phylogenetic group’) (Table S2). The classified RNA viruses that were not previously categorized into a phylogenetic group (18, 26) were kept with their family taxonomic assignments. The majority of the RdRP clusters detected from each ecosystem were taxonomically classified except for those from the invertebrate data set that lacked assignments at the family level (Fig. 2b). We further assessed the taxonomic diversity of the RNA viruses detected from each ecosystem by comparing their relative richness (Fig. 2b). For this analysis, the number of classified families or phylogenetic groups was normalized by the number of classified clusters to represent the taxonomic diversity of the RNA viral communities. Although the California grassland contained the most RdRP clusters (i.e., sequence diversity of phylogenetic markers), the taxonomic diversity of the corresponding RNA viral community was the lowest for this sample set. By contrast, the sequences from thawed permafrost harbored relatively more RNA viral families and phylogenetic groups (Fig. 2b). Consistent with the sequence similarity-based comparisons, we found that some of the RNA viruses were specific to specific sites at a higher taxonomic level (i.e., phylogenetic groups). *Mitoviridae* and Narna-Levi were the most diverse phylogenetic groups with relatively more RNA viral clusters assigned (Fig. S2), and these were primarily detected in the California grassland. Narna-Levi was also the most diverse group in the other grassland soil (‘Kansas_Grassland’). Hepe-Virga and Picorna-Calici groups were the most diverse in invertebrate associated RNA viruses. By contrast, the Tombus-Noda group was the most diverse in thawed permafrost. These results reveal that RNA viruses from thawed permafrost are phylogenetically distinct from those detected in the other sampled terrestrial ecosystems. However, these results also largely reflect the fact that environmental RNA viruses are undersampled and understudied. The unique assemblages of the thawed permafrost RNA viral community compared to the few other environmental habitats studied to date also reflect the environmental conditions that are specific to thawed permafrost and the resulting selection for residing eukaryotic members that are RNA viral hosts.

10.1128/msystems.00582-22.5 FIG S2 Phylogenetic assignments of the RNA viral clusters detected from each ecosystem. The alluvial plot shows the pairings of the RNA viral clusters (‘Viral cluster’) detected from each ecosystem (‘Ecosystem’) to the phylogenetic group assignments (‘Phylogenetic group’). The alluvia were colored by the phylogenetic assignment of each detected permafrost RNA viral cluster. Download FIG S2, PDF file, 1.6 MB. https://doi.org/10.1128/AuthorWarrantyLicense.v1 This is a work of the U.S. Government and is not subject to copyright protection in the United States. Foreign copyrights may apply.

### Potential RNA viral hosts in thawed permafrost.

**FIG 3:**
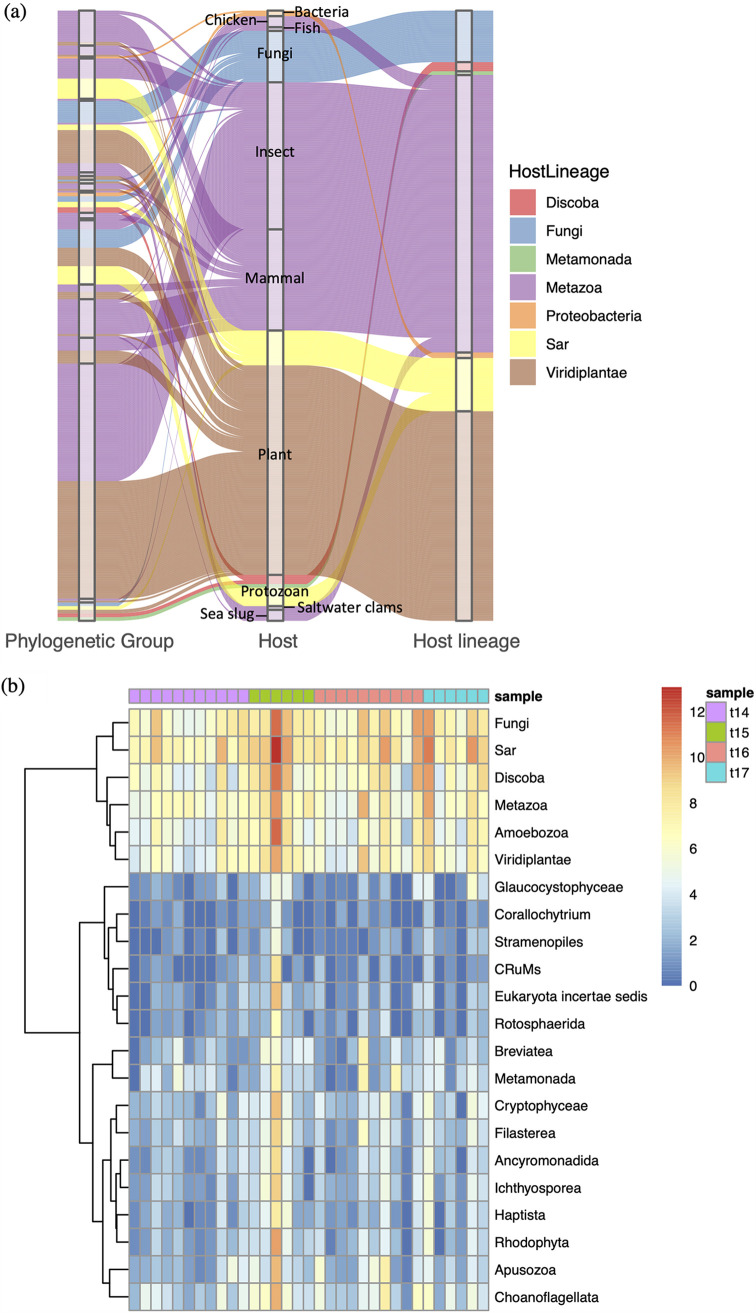
Potential RNA viral hosts in thawed permafrost. (a) Pairings of RNA viral groups and predicted host assignments. The left stratum represents the RNA viral phylogenetic groups detected. The middle stratum shows the general names of known hosts of the detected RNA viruses. The right stratum lists the paired scientific names of the predicted hosts that are specified in (b). The pairings were colored by host lineage assigned. (b) Heatmap illustrating the composition of eukaryotic communities that were detected from 33 thawed permafrost samples that were collected across the following previously published permafrost transects (25): ‘t14’, ‘t15’, ‘t16’, and ‘t17’. The 18S rRNA transcript abundances of the detected eukaryotes were log transformed and color-coded with warmer colors representing higher relative abundances. The eukaryotic groups were clustered by similarity in the distribution patterns across samples.

To identify the potential hosts of the RNA viruses in thawed permafrost, we first established viral-host linkages by retrieving host assignments of the detected RNA viral families from the Virus-Host database (DB) (34). The majority of the RNA viruses targeted eukaryotic hosts, while only a few clusters were paired with prokaryotic hosts (Fig. 3a). The largest proportion of RNA viruses were assigned to hosts within a Metazoan lineage (including insects and mammals), followed by *Viridiplantae*, fungi, and the SAR supergroup (*Stramenopiles*, *Alveolata*, and *Rhizaria*). The potential hosts of the detected RNA viruses were also linked to the dominant groups within the eukaryotic communities (Fig. 3b). One limitation of this approach is that one RNA viral family can infect multiple hosts in some cases. Currently, it is not possible to validate specific viral infections but instead the possible host range is predicted based on current knowledge. Screening of 18S rRNA gene transcripts revealed that the eukaryotes across the permafrost transects were mainly composed of fungi, the SAR supergroup, *Discoba*, *Metazoa*, *Amoebazoa*, and *Viridiplantae* (*Streptophyta* and *Chlorophyta*, including some algae). Another potential limitation of using existing databases to predict hosts of the detected RNA viruses is that our current knowledge of plant viruses is more complete relative to that of fungal viruses. This may partially contribute to the higher representation of plant viruses detected in our data set.

Because the incubations were contained in small soil slurries, the representative Eukarya were presumably microeukaryotes, such as protists and fungi. The finding of transcripts for plants and algae remains enigmatic as the incubations were in the dark. One possibility is that these RNA transcripts were preserved in dead biomass. However, single-stranded RNA is known to be rapidly degraded in soil environments (35), and, thus, the likelihood of retaining free RNA molecules intact during the 97-day incubation is low. Another explanation is that the hosts persisted in a viable state during the incubations. Further research is therefore needed to clarify the underpinning reasons for this finding. Interestingly, a similar eukaryotic community was found in metatranscriptomes of thawed permafrost samples collected in Svalbard, Norway, where *Metazoa*, *Viridiplantae*, SAR supergroup (i.e., members of *Alveolata* and *Rhizaria*), fungi, and *Discoba* (*Excavata* is a clade of *Discoba*) were detected as the main eukaryotic groups (24), which lends support to our findings.

Each of the eukaryotic groups detected (Fig. 3b) were assigned with RNA viruses, except for *Amoebazoa* (Fig. 3a). The lack of detection of *Amoebazoa* could either be due to a deficiency within the database that was used to predict hosts of RNA viruses, or their viruses were either not present, or in low abundances in our incubations. Previously, members of *Amoebazoa* have shown to be infected by giant viruses which are DNA viruses (36), and only one RNA virus has been ascribed/assigned to an Amoebazoan host, to date (37). Therefore, the few RNA viruses that are known to infect *Amoebazoa* may be below the detection limits used for metatranscriptome analyses. The finding of RNA viruses in thawed permafrost that corresponded to dominant eukaryotic groups in the same samples suggests that the recovered RNA viruses influence the permafrost eukaryotic community dynamics. One explanation for this finding may simply be that the dominant eukaryotes have the most viruses.

Redundancy Analysis (RDA) further supported a close linkage between the detected RNA viruses and eukaryotes in thawed permafrost. Forward stepwise model building based on adjusted R^2^ was used to examine the degree to which variation in viral community composition was explained by eukaryotic community composition, environmental variables, and spatial variation based on principal coordinates of neighbor matrices (PCNMs), independently. Eukaryotic community composition best explained viral community composition and supported a final model with four variables (abundance of SAR supergroup, supergroup of Collodictyonids, Rigifilids, and Mantamonas or CRuMs, *Metazoa*, and fungi) fitting the data with an adjusted R^2^ = 0.71. The analysis linking environmental variables to viral community composition supported a final model with one variable (oxygen concentration post-incubation) fitting the data with an adjusted R^2^ = 0.14. Spatial variation based on PCNMs original permafrost sampling location alone was not significantly associated with the RNA viral communities (Data set S1).

10.1128/msystems.00582-22.6 DATA SET S1 Eukaryotic hosts influence RNA viral community assemblages in thawed permafrost. The RDA modeling results are shown for each analysis. Download Data Set S1, DOCX file, 0.02 MB. https://doi.org/10.1128/AuthorWarrantyLicense.v1 This is a work of the U.S. Government and is not subject to copyright protection in the United States. Foreign copyrights may apply.

The eukaryotic communities were significantly influenced by soil physicochemical properties (Data set S1). Modeling to assess the degree to which variation in the eukaryotic community was explained by environmental variables supported a final model with two environmental variables (oxygen concentration post-incubation and soil manganese concentration) fit the data with an adjusted R^2^ = 0.29. Oxygen concentration as an emergent condition post-thaw has also been shown to impact the bioavailability of organic matter (38). Manganese concentrations could reflect broader differences in soil physicochemistry, or could directly influence eukaryotic composition, particularly as manganese is an important cofactor for lignin-metabolic enzymes produced by fungi, which has been reported to shape soil fungal community structure and function (39). As a result, organic matter availability can be shaped by a combination of oxygen and manganese availability, imposing selective pressures on the eukaryotic community (40) and, in turn, the RNA viral composition indirectly.

### Potential RNA viral pathogens in thawed permafrost.

The identified RNA viral families in thawed permafrost included potential plant and animal pathogens. For example, *Tospoviridae* (Fig. 1b) are transmitted between plants by insect vectors (41), have a broad host range of more than 1000 plant species, including economically important crops (42), and cause an estimated annual loss of over USD one billion globally (43, 44). In addition, the *Reoviridae* family includes some plant pathogens, such as rice dwarf virus, rice ragged stunt virus, and rice black-streaked dwarf viruses (45). *Reoviridae*, the largest and most diverse family of double-stranded RNA (dsRNA) viruses (46), were highly represented in our samples, counting for nearly half of abundance estimates of the detected dsRNA viruses (Fig. 1a). Thus, the high abundance of *Reoviridae* may pose a potential threat to plant health in the Arctic as permafrost thaws. We also detected sequences corresponding to *Nairoviridae* and *Hantavirus* families (Fig. 1b) which contain members that can cause serious human diseases, such as hemorrhagic fever (47). It is noteworthy that these viral families also include members that infect *Amoebazoa* (37, 48) which pose less of a threat to humans. It was disconcerting to find viruses in the *Coronaviridiae* family because it includes strains that can be pathogenic to humans, including the SARS-CoV-2 strain responsible for the COVID-19 pandemic. In this case, the detected viruses shared high sequence similarities to a feline infectious peritonitis virus (strain 79-1146, RdRP: Q98VG9.2) and to an avian infectious bronchitis virus (strain M41, RdRP: P0C6Y3.1). No evidence to date shows that the two detected strains can infect humans based on the Immune Epitope Database and Analysis Resource (http://www.iedb.org/home_v3.php). However, based on these data, it is still not possible to determine if there are other unclassified viral sequences that could represent infectious human viral pathogens. By contrast, known bacterial pathogens (e.g., Bacillus anthracis) have emerged from thawed permafrost (49) as well as fungal pathogens that can infect plants (e.g., Aspergillus) (50). Fragments of DNA viral pathogens have also been detected in permafrost without direct evidence of activity (51). Our results also suggest that genomes or fragments of RNA viruses, including potential pathogens, can be recovered from thawing permafrost. Further study is needed to confirm infectivity, activity, and pathogenicity in modern hosts (11).

Mycoviruses, which infect fungi with symptomless (cryptic or latent symptoms) infections (52, 53), were also observed in our samples, including *Partitiviridae*, *Reoviridae*, *Totiviridae*, *Hypoviridae*, *Mitoviridae*, and *Narnaviridae* (Fig. 1a and c). Mycoviruses from families *Partitiviridae* and *Totiviridae* have co-evolved with their respective hosts and evidence suggests they may have been integrated into host genomes (54). For example, Partitivirids (viruses belonging to *Partitiviridae*) are a family of cryptic viruses (55, 56) whose transmission occurs vertically during cell division or horizontally through cell-cell contacts. They have a virion phase as encapsidated viruses within the host, but they do not appear to have an extracellular transmission stage (57). Thus, these mycoviruses may be preserved in permafrost in their host cells.

### Functional annotations of RNA viruses.

Functional genes were predicted and annotated from the RNA viral sequences to investigate how RNA viruses in thawed permafrost might influence their hosts and ecology of the ecosystem. To date, there is no bioinformatic tool tailored to annotate putative auxiliary metabolic genes (AMGs) in RNA viruses (58, 59). Therefore, we applied stringent annotation criteria to assign functional genes from RNA viral contigs that passed our quality thresholds (RdRp, max. E-value of 2.50E-12, min. bit score of 50.3; sequence similarity to NCBI RNA viral genomes, max. E-value of 9.99E-06, min. bit score of 56.5) (Table S2). Putative AMGs that were predicted to be on RNA viral families that are known to integrate into host genomes (e.g., retroviruses, *Flaviviridae* and *Totiviridae*) (54, 60) were excluded from our final list (Table S3). A range of gene categories were classified that encoded conserved RNA viral proteins, including viral structural proteins (e.g., viral coat/core proteins and pilin-like proteins), proteins for viral replication, and assembly (e.g., DNA and RNA polymerases, and nucleotide binding proteins) (Table S3). These core proteins were not considered to be AMGs. However, several AMGs that are not directly required for viral production were also detected. Unlike the conserved proteins, the majority of the putative AMG proteins were sparsely detected, and most were classified as incomplete proteins (lacking start and/or stop codons) (Table S3). Examples of putative AMGs include those encoding enzymes for carbon utilization, and host-related metabolism (e.g., biosynthesis and carbohydrate metabolism) (Table S3).

10.1128/msystems.00582-22.3 TABLE S3 Functional annotation of detected RNA viral contigs. Information for each predicted protein from RNA viral contigs and their functional annotations with statistics. Download Table S3, XLSX file, 0.06 MB. https://doi.org/10.1128/AuthorWarrantyLicense.v1 This is a work of the U.S. Government and is not subject to copyright protection in the United States. Foreign copyrights may apply.

**FIG 4:**
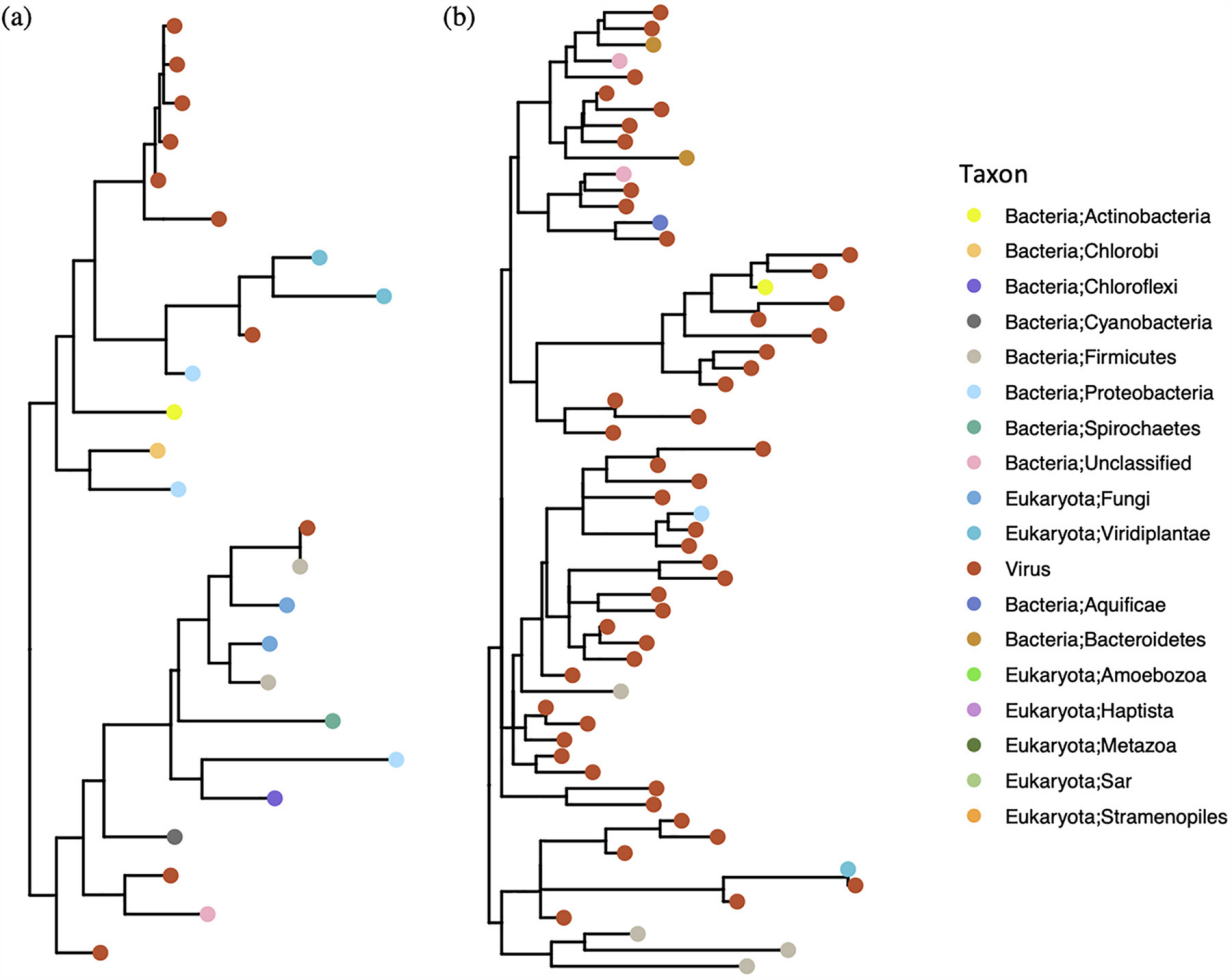
Phylogenetic trees of putative AMG proteins encoded by RNA viruses in thawed permafrost. Phylogenetic trees of (a) polygalacturonase and (b) cell wall hydrolase were constructed using multiple sequence alignments of the corresponding proteins detected from the RNA viral contigs and the reference sequences of non-viral genomes deposited in NCBI NR databases. The leaves of the trees are colored by the taxonomic assignments of the sequences. The sequences detected from RNA viral contigs in this study are colored in red.

Two AMG transcripts were relatively more prevalent in the thawed samples collected from the permafrost transect sample set: polygalacturonase 1 (PG1) and cell wall hydrolase (Table S3). To our knowledge, this is the first time that PG1 has been observed in RNA viruses. Polygalacturonase 1 is an enzyme which hydrolyzes the alpha 1, 4 glycosidic bonds between galacturonic acid residues, effectively degrading the adhesive pectic bonds of the middle lamella and cell wall (61, 62). PGs are also widely distributed in domains of life and are present in both pathogens and non-pathogens. The PG1 sequences detected from RNA viruses in our samples shared conserved regions with the reference PG sequences (Data set S2), and formed a separate clade from those in prokaryotes and eukaryotes (Fig. 4a). The PG1 genes were detected on contigs assigned to *Rhabdoviridae* and *Phasmaviridae*, with Eukaryotes as hosts. These RNA viruses, unlike the ones that are passed via host meiosis, are known to replicate cytoplasmically and release from hosts via budding (63), which may result in the unique sequence features of viral PG1. The majority of the detected PG1 sequences were partial (38 out of 39 genes) which could either be due to incomplete assemblies, or the transcripts were naturally truncated. Truncated PG transcripts have previously been reported to inhibit the expression of endogenous genes in plants (64). Alternatively, the PG1 genes on RNA viruses may be involved in degradation of plant cell walls or deposited plant detritus. Further investigations are therefore needed to clarify the ecological roles of PG1 genes in soil RNA viruses.

10.1128/msystems.00582-22.7 DATA SET S2 Conserved regions of functional genes across taxa. Download Data Set S2, DOCX file, 0.02 MB. https://doi.org/10.1128/AuthorWarrantyLicense.v1 This is a work of the U.S. Government and is not subject to copyright protection in the United States. Foreign copyrights may apply.

We also observed a relatively high abundance of AMG transcripts corresponding to cell wall hydrolases in *Leviviridae*- and *Reoviridae*-like RNA viruses (Table S3). Unlike viral PG1, these sequences were more diverse (more representative sequences after clustering), shared conserved regions with reference sequences (Data set S2), and many formed tighter clusters with prokaryotic and eukaryotic versions (Fig. 4b and c). These results suggest that the cell wall hydrolase sequences were subject to different sequence diversification selection pressures when compared to PG1.

RNA viruses are known to have smaller genome sizes than DNA viruses and mainly pack genetic material that is essential for their own propagation (65). Therefore, the detection of these AMGs in RNA viruses suggests that they were selected to aid in viral infection. In addition, although there is increasing evidence of exchange of genes between viruses and cells (66–69), little is known about the functions of potentially exchanged AMGs. Therefore, more empirical work is needed to validate the functions of the putative AMGs in RNA viruses. However, from an ecological perspective, prevalence of specific types of AMGs on viruses could reflect selection pressure by the ecosystem to maintain or enrich the representative functions (70, 71).

### Summary.

This study reveals the unique composition of RNA viruses in thawed permafrost compared to other terrestrial environments studied to date and their potential hosts, with most hosts being eukaryotic taxa. Because this incubation experiment simulated permafrost thaw, the RNA viral sequences detected can be used as a reference for predicting preserved and potentially active RNA viruses, including pathogens, that can emerge from permafrost under a warming climate. As revealed by the modeling results, the detected RNA viral community composition in thawed permafrost was most closely related to the dominant eukaryotic taxa in the samples that were their potential hosts. The finding of auxiliary metabolic gene sequences in RNA viruses that may be involved in carbon cycling and viral-host interactions opens a window for new exploration into the ecological roles of RNA viruses in permafrost as it thaws.

## MATERIALS AND METHODS

### Permafrost soil sampling and incubation.

Permafrost cores were collected from the Caribou Poker Creek Research Watershed near Fairbanks, AK in August 2015. A total of 33 permafrost samples along four parallel transacts were collected and preserved frozen on dry ice during shipment to the Pacific Northwest National Laboratory and subsequently stored at −20°C as intact cores (detailed sample locations in Table S1). The top 6 cm of each core was removed and the remaining 7 to 14 cm section of frozen permafrost soil was processed as previously described (25). A comprehensive suite of chemical data was collected from the samples as previously described (25) (Table S1). The frozen permafrost samples were individually homogenized in purpose-built stainless steel sample homogenizers (akin to a mortar and pestle), which were alcohol washed, flame-sterilized, and cooled on dry ice, and aseptically dispensed (25 to 50 g) into 50 mL Falcon tubes. The samples were then incubated at 4°C in the dark to mimic natural permafrost thaw conditions that occur in the dark ~1 to 2 m below the seasonally thawed active layer. A 97-day incubation period was used to mimic the ~3 month deepening of the active layer as the permafrost thaws, and for the microbial populations to have time to adapt and increase in biomass. Because the samples were incubated without shaking, this provided an opportunity for a redox gradient to form, similar to what occurs in nature. Following incubation, oxygen concentrations were measured through the vertical profile of the incubate sample using O_2_ microsensors (Unisense), and the samples were immediately flash frozen in liquid nitrogen.

RNA was extracted from frozen duplicate ~2 g samples collected from the bottom 30 mm section of each incubated sample using the MoBio RNA PowerSoil Total RNA isolation kit (MoBio Laboratories). Duplicate extractions were done strictly to get enough RNA for sequencing, and these duplicate extractions were performed consistently for each sample to ensure the RNA pools represented extracts from the same amount of total soil for each sample (i.e., the RNA for each sample came from ~4 g soil total). To guard against contamination, all manipulations of frozen samples were performed using flame-sterilized steel implements, on heat sterilized aluminum foil-covered surfaces (450°C for 8 h) in a biological safety cabinet. Samples were homogenized in purpose-built stainless steel sample homogenizers, which were alcohol washed, flame-sterilized, and cooled on dry ice. Samples were poured directly from the homogenizers into sterile Falcon tubes and stored at −80°C until extraction. Samples were transferred from Falcon tubes into extraction tubes using a flame-sterilized scoopula. Individual RNA extractions yielded between 800 ng and 6500 ng of RNA per extraction, and pools of duplicate extractions contained between 1900 ng and 11970 ng RNA total, as determined by Qubit RNA HS kit assays (Thermo Fisher Scientific), which provided sufficient RNA for sequencing.

### Metatranscriptome sequencing and *de novo* assembly.

Metatranscriptomic sequencing was completed on Illumina HiSeq2500 sequencing platform at Argonne National Laboratory (Lemont) (sequencing statistics in Table S1). The raw metatranscriptomic reads were trimmed and quality controlled using Trimmomatic (v0.33) (score > 30 and length > 36 bases). The remaining reads were mapped to PhiX genome using Burrows-Wheeler Aligner (BWA, v0.7.17) and the exact matches were removed to avoid the contamination from PhiX that was used as a quality and calibration control of sequencing runs. The quality-filtered metatranscriptomic reads were then *de novo* assembled by rnaSPAdes (v3.13.0) using default settings.

### Identification of RNA viral contigs from metatranscriptome assemblies.

As RNA viral (segmented) genomes vary in size by 1 order of magnitude (65), we did not limit the searching only against long contigs but applied stringent matching criteria to screen for all the potential RNA viral contigs to provide a high coverage of RNA viral diversity. RNA viral contigs were identified by searching for the marker gene, RdRp, using a set of curated Hidden Markov Models (HMMs) (E-value cut-off 1 × 10^−5^, bit score cut-off 50, details in ‘Data and code availability’), complemented with the whole sequence similarity searches against NCBI RNA viral genomes (E-value cut-off 1 × 10^−5^, bit score cut-off 50). When the assembly was too short to detect a RdRp gene, the identified RNA viral contig inherited the taxonomy assignment of the qualified hit in NCBI RNA viral genomes and the RdRp gene from the qualified hit genome was extracted to represent the phylogenetic placement of the short RNA viral assembly. Taxonomy assignment of the identified RNA viral contigs was based on RdRp gene detected or assigned. As the RNA viral contigs were classified based on RdRp, RNA viruses without the RdRp gene, such as retroviruses and plant viroids (72), were not included in this study.

### Phylogenetic reconstruction of permafrost RNA viruses.

All the retrieved RdRp amino acid sequences were de-replicated at 99% amino acid identity using CD-hit (73). The longest RdRp sequences of each cluster were selected as the representatives. According to the assigned taxonomy annotation, the RdRp sequences were separated into three groups representing double-stranded (ds), negative single-stranded (-ss), and positive single-stranded (+ss) RNA viruses. Within each group, the representative sequences were aligned together with a DNA-directed RNA polymerase from *Alphaproteobacteria* (WP_012231479.1) as an outgroup using MAFFT (Multiple Alignment with the Fast Fourier Transform, v7). The multiple sequence alignment (MSA) was manually inspected and adjusted in Jalview (v2.11.1.4). The MSA was then used to construct a phylogenetic tree for each group of RNA viruses that was rooted by the outgroup using FastTree (v2.1.10).

### Host assignment and abundance estimation of RNA viruses in thawed permafrost.

As the majority of detected RNA viral contigs were classified and assigned to a Family based on RdRp, we can predict their hosts by leveraging the Virus-Host DB (https://www.genome.jp/virushostdb/) with the virus-host pairing information manually curated from NCBI Virus, Refseq, UniProt, and related literature. For a relatively conservative prediction, host lineage was assigned to Phylum or Superphylum level.

The average read coverages of the detected RNA viral contigs were calculated to represent their genomic coverage. To avoid double counting, only the quality-filtered forward metatranscriptomic reads were mapped to the *de novo* assemblies from each sample. The mappings were filtered with stringent criteria of identity greater than 95% and coverage higher than 80% by BamM (v1.7.3, bamm make and bamm filter). We then used samtools (v1.9, samtools depth) to calculate the reads coverage per base for each assembled contig. The abundances of the identified RNA viral contigs were estimated by the average base-coverage of the assembly after normalizing by the total quality-filtered forward reads across all the samples.

### A cross-study comparison in assembling RNA virosphere.

To compare RNA viruses detected in thawed permafrost to those previously detected in other terrestrial habitat types, we collected the RdRp protein sequences from metatranscriptome data from three published studies; one from invertebrates (26) and two from grassland soils (18). RdRp sequences retrieved from the three reference studies and from our study were first de-replicated at 99% amino acid identity. The de-replicated RNA viral contigs from the four studies were then pooled together and further clustered at 70% of amino acid identity. The clusters that were shared and unique to each RNA virosphere were counted. Non-singleton clusters were visualized in a network (Fig. S1) with nodes representing the clustered RdRp sequences colored by type of biome detected. We also calculated the number of unique families or phylogenetic groups relative to the number of classified clusters and defined this as ‘relative richness’ in order to compare the taxonomic diversity of RNA viruses across the ecosystems.

### Annotating functional potentials of the detected RNA viruses.

Genes were predicted from the identified RNA viral contigs and translated into protein sequences by Prodigal (v2.6.3). The protein sequences were then annotated by a collection of databases using *hmmsearch* (HMMER 3.3) with the parameters as previously described (7). In brief, annotation database including three non-viral databases (i.e., EggNOG bacterial and archaeal databases [74], the functional ontology assignments for metagenomes database or FOAM [75]), and four viral databases (i.e., EggNOG viral database, Nucleo-Cytoplasmic Viruses Orthologous Groups or NCVOG [76], and two curated viral profile HMMs based on NCBI virus and Pfam database [77]). The functional annotation of the searched protein was assigned by the database that had the highest bit score. The annotated gene categories across multiple databases were manually inspected and combined.

Due to their relatively high prevalence in our samples, polygalacturonase and cell wall hydrolase were selected to construct protein trees and to assess their sequence relatedness to the close hits in the public database. The protein sequences were first dereplicated at 99% identity using CD-HIT (73) and searched against the clustered NR database (accessed on May 6th 2022) to retrieve the top 10 hits of each query sequence. For each gene type, the subject sequences were dereplicated at 70% of identity (CD-HIT) to capture the sequence diversity. The functional annotations of the subject sequences were consistent with the query sequences except for ones that were labeled as hypothetical. The hypothetical proteins were annotated using the same databases as mentioned above. The verified subject sequences were aligned with the query viral sequences by MAFFT (v7) using default settings. The multiple sequence alignments were used to construct the phylogenetic trees using maximum-likelihood method via FastTree (v2.1.10).

### Screening and annotating 18S rRNA transcript reads.

The 18S rRNA transcript reads that were extracted from the metatranscriptomes were analyzed to determine eukaryotic community compositions. The quality-filtered forward metatranscriptomic reads were first mapped to a well-curated set of reference sequences in the Silva Database (silva-euk-18s-id95) using BamM (v1.7.3, bamm make, https://github.com/Ecogenomics/BamM) and filtered by percent identity higher than 0.95 and percent alignment greater than 0.80 (BamM v1.7.3, bamm filter). The transcript abundances of members of the Eukarya were estimated by the average base coverage of the mapped 18S rRNA reference sequences (samtools v1.9, samtools depth, http://www.htslib.org/doc/) normalized by the total counts of reads per sample.

### Modeling drivers of community variations.

An RDA modeling approach was used to evaluate how much variation in the thawed permafrost RNA viral communities could be explained by eukaryotic community composition, environmental variation, and spatial variables (PCNMs) using the *vegan* package in R. Forward stepwise model building based on adjusted R^2^ was used to determine the variables that were most important for explaining variation in the log (x + 1) transformed values of viral community composition using three data sets independently as variables: (i) log (x + 1) transformed values for eukaryotic community composition, (ii) standardized environmental variables, and (iii) PCNMs derived from GPS coordinates of original sampling locations. The same modeling approach was used to determine relationships between log (x + 1) transformed values of eukaryotic community composition and standardized environmental variables. All models were run with 200 permutations.

### Data availability.

The raw metatranscriptome data were deposited to NCBI with BioProject ID PRJNA852283. The RdRP profile HMMs and the R code used for statistical analysis and data visualization are available at https://github.com/Ruonan0101/PermafrostVirome_PNNL.
