# Supplementary figures and images for: RNA Viruses Linked to Eukaryotic Hosts in Thawed Permafrost
Source: mSystems. 2022 Dec 1;7(6):e00582-22. doi: 10.1128/msystems.00582-22 (PMC9765123; doi:10.1128/msystems.00582-22)

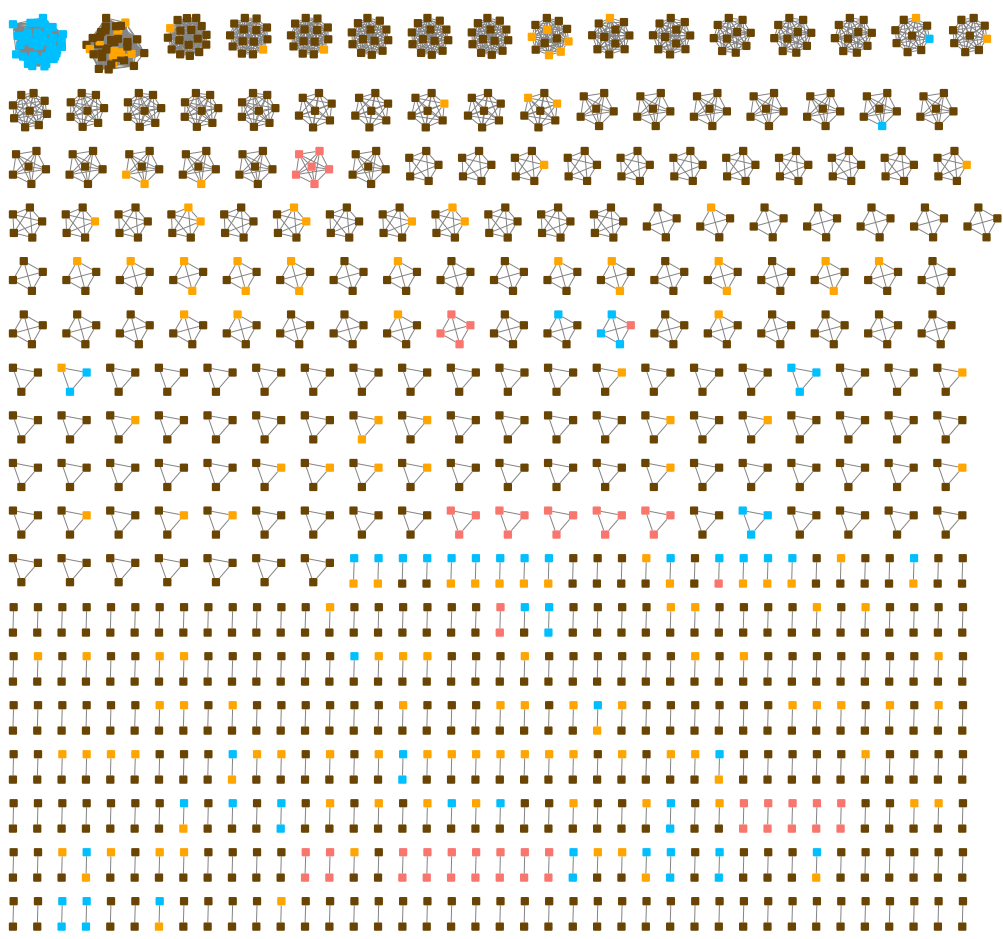

Supplement: FIG S1 [file msystems.00582-22-s0004.pdf]
