# Supplementary material for: RNA Viruses Linked to Eukaryotic Hosts in Thawed Permafrost
Source: mSystems. 2022 Dec 1;7(6):e00582-22. doi: 10.1128/msystems.00582-22 (PMC9765123; doi:10.1128/msystems.00582-22)
Supplement: FIG S2 [file msystems.00582-22-s0005.pdf]

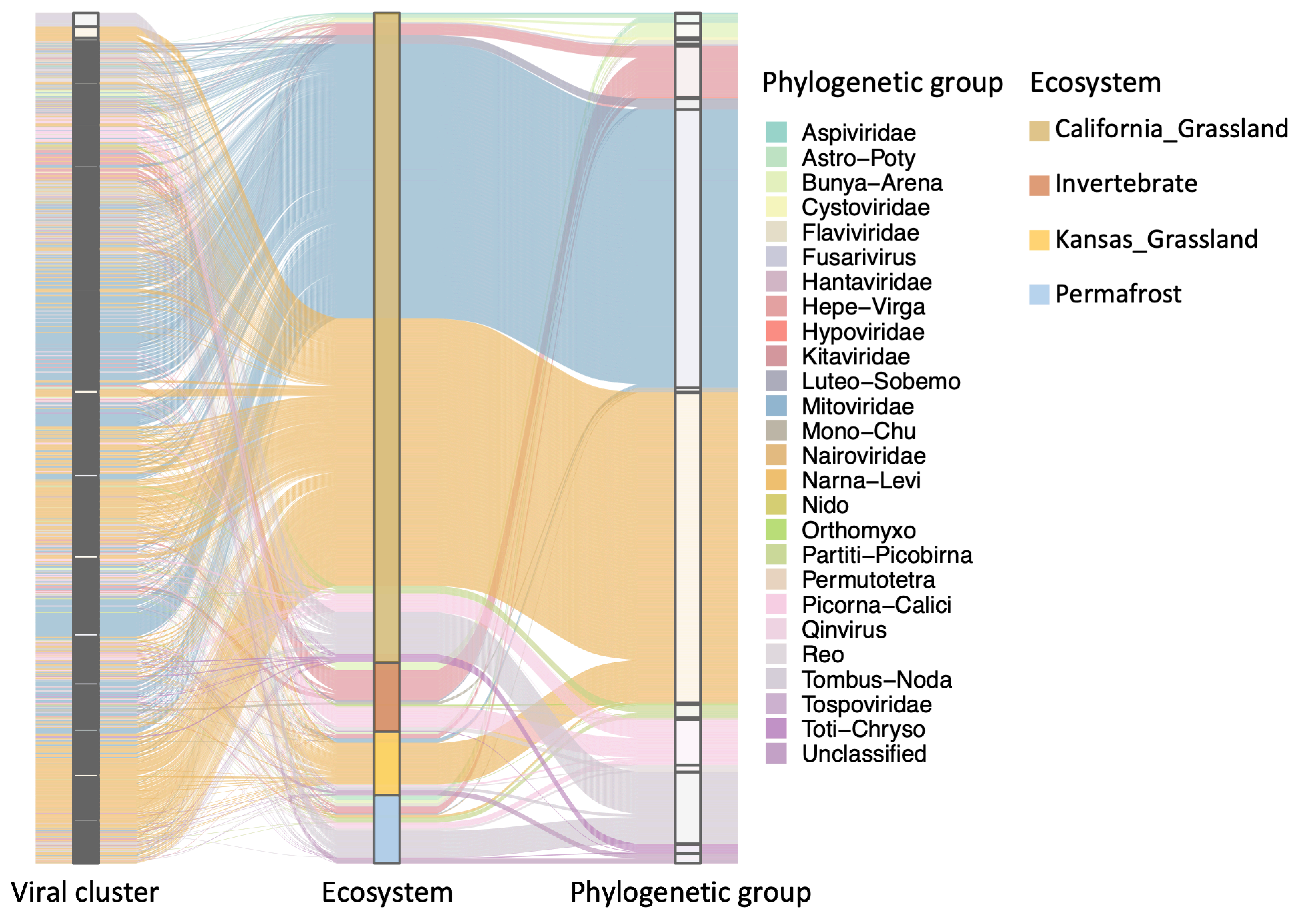
Phylogenetic group
Aspiviridae
Astro–Poty
Bunya–Arena
Cystoviridae
Flaviviridae
Fusarivirus
Hantaviridae
Hepe–Virga
Hypoviridae
Kitaviridae
Luteo–Sobemo
Mitoviridae
Mono–Chu
Nairoviridae
Narna–Levi
Nido
Orthomyxo
Partiti–Picobirna
Permutotetra
Picorna–Calici
Qinvirus
Reo
Tombus–Noda
Tospoviridae
Toti–Chryso
Unclassified
Ecosystem
California_Grassland
Invertebrate
Kansas_Grassland
Permafrost
Viral cluster
Ecosystem
Phylogenetic group
