# Supplementary material for: RNA Viruses Linked to Eukaryotic Hosts in Thawed Permafrost
Source: mSystems. 2022 Dec 1;7(6):e00582-22. doi: 10.1128/msystems.00582-22 (PMC9765123; doi:10.1128/msystems.00582-22)
Supplement: DATA SET S1 [file msystems.00582-22-s0006.docx]

**Supplementary Data 1**

**RNA Viruses Target Diverse Eukaryotic Hosts in Thawed Permafrost**

Ruonan Wu^1 *^, Eric M. Bottos^2^, Vincent G. Danna^1^, James C. Stegen^1^, Janet K. Jansson^1^, Michelle R. Davison^1^

1. Earth and Biological Sciences Directorate, Pacific Northwest National Lab, Richland, WA 99354, USA
2. Faculty of Science, Thompson Rivers University, Kamloops, Canada

*Corresponding author

E-mail: [ruonan.wu@pnnl.gov](mailto:ruonan.wu@pnnl.gov)

*An analysis to determine how well the viral community composition is explained by the eukaryotic variables shows the Sar, CRuMs, Metazoa, and Fungi are the most important eukaryotic variables explaining viral composition:*

viralEukmodel<-ordiR2step(viralEukmodel1, viralEukmodel2, perm.max=200)

Step: R2.adj= 0.70561

Call: Tlogviral ~ Sar + CRuMs + Metazoa + Fungi

R2.adjusted

<All variables> 0.7523092

+ Haptista 0.7119808

+ Corallochytrium 0.7117793

+ Amoebozoa 0.7094225

+ Rhodophyta 0.7085599

+ Glaucocystophyceae 0.7084520

+ Apusozoa 0.7083598

+ Metamonada 0.7083100

+ Breviatea 0.7072936

+ Viridiplantae 0.7064526

+ Eukaryota.incertae.sedis 0.7063884

<none> 0.7056100

+ Filasterea 0.7054151

+ Cryptophyceae 0.7050112

+ Ancyromonadida 0.7044209

+ Stramenopiles 0.7037693

+ Ichthyosporea 0.7020493

+ Rotosphaerida 0.7018791

+ Discoba 0.7017829

+ Choanoflagellata 0.7003958

Df AIC F Pr(>F)

+ Haptista 1 100.63 1.6193 0.084 .

---

Signif. codes: 0 ‘***’ 0.001 ‘**’ 0.01 ‘*’ 0.05 ‘.’ 0.1 ‘ ’ 1

*An analysis to determine how well the viral community composition is explained by the environmental variables shows oxygen is the most important environmental variable explaining viral composition:*

viralEnvmodel<-ordiR2step(viralEnvmodel1, viralEnvmodel2, perm.max=200)

Step: R2.adj= 0

Call: Tlogviral ~ 1

Df AIC F Pr(>F)

+ Oxygen 1 133.2 6.3089 0.004 **

---

Signif. codes: 0 ‘***’ 0.001 ‘**’ 0.01 ‘*’ 0.05 ‘.’ 0.1 ‘ ’ 1

Step: R2.adj= 0.142296

Call: Tlogviral ~ Oxygen

R2.adjusted

<All variables> 0.4015399

+ Mn 0.1903004

+ Lactate 0.1770458

+ Acetate 0.1751670

+ Fe.II. 0.1743791

+ Water 0.1634364

+ Sand 0.1631415

+ Mg 0.1622194

+ ALD 0.1609674

+ pH 0.1485631

+ Mud 0.1444529

<none> 0.1422960

+ P 0.1415410

+ NO3 0.1362912

+ Formate 0.1340413

+ Cl 0.1315732

+ Cu 0.1315140

+ S 0.1299790

+ Gravel 0.1283681

+ C 0.1281027

+ N 0.1278659

+ SO4 0.1274403

+ Fe.Tot. 0.1264658

Df AIC F Pr(>F)

+ Mn 1 132.22 2.8379 0.054 .

---

Signif. codes: 0 ‘***’ 0.001 ‘**’ 0.01 ‘*’ 0.05 ‘.’ 0.1 ‘ ’ 1

*An analysis to determine how well the viral community composition is explained by the PCNMs shows no significant relationship between viral composition and PCNMs:*

> viralPCNMmodel<-ordiR2step(viralPCNMmodel1, viralPCNMmodel2, perm.max=200)

Step: R2.adj= 0

Call: Tlogviral ~ 1

R2.adjusted

+ PCNM7 0.0345667128

+ PCNM17 0.0271953257

+ PCNM18 0.0144775847

+ PCNM13 0.0130880974

+ PCNM8 0.0002610659

<none> 0.0000000000

+ PCNM19 -0.0013355336

+ PCNM10 -0.0014447041

+ PCNM4 -0.0017445689

+ PCNM15 -0.0052552883

+ PCNM9 -0.0058790746

+ PCNM5 -0.0091947494

+ PCNM12 -0.0135838494

+ PCNM2 -0.0137570095

+ PCNM1 -0.0139252275

+ PCNM11 -0.0152184521

+ PCNM6 -0.0187235757

+ PCNM3 -0.0208996750

+ PCNM20 -0.0211493968

+ PCNM16 -0.0213942750

+ PCNM14 -0.0257895129

<All variables> -0.2575741194

*Taking only the variables that were significant in the individual models and combining them to determine how well the viral community composition is explained by these variables together (basically, does oxygen explain additional variation in the viral composition data that is not explained by the eukaryotic variables). Including oxygen with the eukaryotic variables did not improve the model.*

Signif. codes: 0 ‘***’ 0.001 ‘**’ 0.01 ‘*’ 0.05 ‘.’ 0.1 ‘ ’ 1

Step: R2.adj= 0.6798359

Call: Tlogviral ~ Sar + CRuMs + Metazoa

R2.adjusted

+ Fungi 0.7056100

<All variables> 0.7010237

<none> 0.6798359

+ Oxygen 0.6746675

*Finally, how well do environmental variables explain variation in the eukaryotic community composition? Oxygen was the most important variable, and a combination of oxygen and manganese were the most important variables, in explaining eukaryotic composition.*

Step: R2.adj= 0.285567

Call: logeuk ~ Oxygen + Mn

R2.adjusted

<All variables> 0.5252080

+ Fe.II. 0.3247958

+ pH 0.3064279

+ S 0.2999458

+ Lactate 0.2886275

+ N 0.2869751

+ C 0.2864404

<none> 0.2855670

+ SO4 0.2828069

+ Mud 0.2795296

+ Sand 0.2782313

+ NO3 0.2781297

+ ALD 0.2733153

+ Water 0.2730478

+ Formate 0.2724092

+ Cu 0.2718224

+ Mg 0.2717450

+ Acetate 0.2712759

+ Cl 0.2704604

+ P 0.2676979

+ Gravel 0.2674644

+ Fe.Tot. 0.2673707

Df AIC F Pr(>F)

+ Fe.II. 1 130.57 2.743 0.08 .

---

Signif. codes: 0 ‘***’ 0.001 ‘**’ 0.01 ‘*’ 0.05 ‘.’ 0.1 ‘ ’ 1
