# Supplementary material for: RNA Viruses Linked to Eukaryotic Hosts in Thawed Permafrost
Source: mSystems. 2022 Dec 1;7(6):e00582-22. doi: 10.1128/msystems.00582-22 (PMC9765123; doi:10.1128/msystems.00582-22)
Supplement: DATA SET S2 [file msystems.00582-22-s0007.docx]

**Supplementary Data 2**

**RNA Viruses Target Diverse Eukaryotic Hosts in Thawed Permafrost**

Ruonan Wu^1 *^, Eric M. Bottos^2^, Vincent G. Danna^1^, James C. Stegen^1^, Janet K. Jansson^1^, Michelle R. Davison^1^

1. Earth and Biological Sciences Directorate, Pacific Northwest National Lab, Richland, WA 99354, USA
2. Faculty of Science, Thompson Rivers University, Kamloops, Canada

*Corresponding author

E-mail: [ruonan.wu@pnnl.gov](mailto:ruonan.wu@pnnl.gov)

**Conservative regions of the multiple sequence alignment**

**###viral sequences are with ‘exp_’ in the header and the reference sequences are labeled with ‘ref_’)**

**Polygalacturonase sequences**

>exp_PG_t1480mBot_S03RuonanNODE_140986_length_287_cov_10.243902_g139970_i0_1

HRALLRESGAHCAIFPTAASRRSLDRVSVPVWLVILS

>exp_PG_t15104mBot_S031RuonanNODE_54174_length_389_cov_0.000000_g53832_i0_1

HAALLRQGFPHCAKFPTAASRRSLDRVSVPVWLVILS

>exp_PG_t1632mBot_S012RuonanNODE_27223_length_473_cov_6.342857_g26145_i0_1

HAAWLDQACAHCPIFPTAASRRSLGRVSVPMWLIILS

>exp_PG_t1592mBot_S010RuonanNODE_51221_length_342_cov_14.716895_g50656_i0_1

HAAWLHQACAHCAIFPTAAPRRSLDRVSVPVWLVILS

>ref_PG_TBR59005.1

HAVLLRQAFAHCGKFPTAASRRSLDRVSVPVWLIILS

>exp_PG_t1660mBot_S022RuonanNODE_272_length_1005_cov_13.306122_g234_i1_1

HAALLRQGFPHCAIFLTAASRRSLDRVSVPVWLIILS

>ref_PG_CEE24912.1

HAAWLDQACAHCPIFPTAASRRSLDRVSVPVWLIILS

>exp_PG_t1424mBot_S08RuonanNODE_92332_length_301_cov_7.151685_g91448_i0_1

HAAWLHQACAHCAIFPTAASRRSLDRVPVPVWLVILS

>ref_PG_AAG12204.1

HAALLRQAFAHCAIFLTAASRRSLDRVSVPMWLIILS

>exp_PG_t15104mBot_S031RuonanNODE_15720_length_468_cov_0.110145_g15465_i0_1

HAAWLHQACAHCAIFPTAASRRSLDRFSVPVWLFILS

>exp_PG_t1580mBot_S013RuonanNODE_37165_length_411_cov_368.138889_g35840_i4_1

HAALLHQACAHCAIFPTAASRRSLDRVSVPVWLVILS

>exp_PG_t1532mBot_S023RuonanNODE_12673_length_428_cov_0.727869_g11581_i0_1

HAAWLHQACAHCAIFPTAASRRGLDRVSVPVLLVILS

>exp_PG_t16104mBot_S021RuonanNODE_1547_length_622_cov_1.452906_g1435_i1_1

HAALLHQTCVHCGRFPTAAS-----------------

>ref_PG_CBL49508.1

HAALLHQTCVHCGRFPTAASRRSLGRVSVPMWPISLS

>ref_PG_CDB39267.1

HK-------------PSSLTRHGWVRVAS---IAQYS

>ref_PG_AGO88040.1

HAALLRQGFPHCARFPTAASRRSLGRISIPVWLIILS

>ref_PG_RGB21528.1

HAALLHQAFAHCGRFPTAASRRSLGRVSVPVWPITLS

>ref_PG_GIR91464.1

HAASLHQAFAHCARFPAAASRRSGGRVSVPLWLVKLS

>ref_PG_GAN11782.1

HAALLRQAFAHCGRFPTAASRRSLGRVSVPVWPFTLS

>ref_PG_SOX55400.1

HAAWLHQACAHCAIFPTAASRRSLGRISVPVWPDTLS

>ref_PG_CDN41090.1

HAALLRQTCVHCGRFPTAASRRSLGRVSVPVWPITLS

>ref_PG_EPF26580.1

HAASLRQGFPHCGIFLPAASRRSLGRISVPMCPSALS

>ref_PG_CDG40808.1

HAALLDQAFAHCPIFPTAASRRSLGRVSVPVWLIILS

>ref_PG_KAF1857276.1

HAALLDQACAHCPIFPTAASRRSLGRVSVPVWLIILS

>ref_PG_KAF1857267.1

HAALLDQACAHCPIFPTAASRRSLGRVSVPVWLIILS

**Cell wall hydrolase**

>ref_CellWallHydrolase_EAY29055.1/1-212

VQPGPLVLRSGPLKYLTPTTDRDRTVSRRSEPSSRATLMGEQPNPWNLLQLQDVTSRHRGAKPPRRCEL

>ref_CellWallHydrolase_EEP60269.1/1-112

PHPGPLVLRMAPLKYPAPAADRDRTVSRRSEPSSRAALMGEQPNPWDLLQPQDATSRHRGAKPRRRCGL

>ref_CellWallHydrolase_EQG62050.1/35-137

VHPGPLVLRTGLLKFPTPATDRDRTVSRRSEPSSRTTLMGEQPNPWDLLQPQDVMSRHRGAKPPRRCGL

>ref_CellWallHydrolase_AGV54820.1/16-365

VLPGPLVLGKGPLNALTPTPDMDRTVSRRSEPSSRTALMGEQPNPWNILQPQVAKSRHRGAKPSRRCEL

>ref_CellWallHydrolase_CKF24369.1/1-252

VHPGPLVLRTAPLKFPTPTTDRDRTVSRRSEPSSRTALMGEQPNPWDRLQPQDAMSRHRGAKPPX----

>ref_CellWallHydrolase_KPC97162.1/1-225

VHPGPLVLRTAPLKFPPPATDRDRTVSRRSEPSSRTALMGEQPNPWDRLQPQDAMSRHRGAKPPVDVDS

>ref_CellWallHydrolase_CWM28216.1/1-216

VHSGPLVLGAAPVKLPTPTADRDQTVSRRFKPSSRTTLNGEQPYPWDRLQPQDVMSRHRGAKLRRRYEL

>ref_CellWallHydrolase_ANA07971.1/1-136

VHLGPLVLETTPLKPPTPMVDRDRTVSRRSEPSSRTALMGEQPNPWDLLRPQDATSRHRGAKPFRRCGL

>ref_CellWallHydrolase_AOE06232.1/25-156

VHLGPLVLKTDPRKFLTPTTDRDRTVSRRSEPSSRATLMGEQPNPWDLLQPQDVTSRHRGAKPPRRYEL

>ref_CellWallHydrolase_SPT75802.1/1-155

VRPGPLVLGTALFKFPARAADRDRTVSRRSEPSSRAALMGEQPNPWDLLQPQDATSRHRGAKPCRRYEL

>ref_CellWallHydrolase_SUC37659.1/1-222

VHHGPLVLVAAPRKTPTPTIDRDRTVSRRSEPSSRATLMGEQPNPWDLLQPQDVTSRHRGAKPPRRYEL

>ref_CellWallHydrolase_CAC9422096.1/1-142

VHPGPLVLRTAPLKFPTPTTDRDRTVSRRSEPSSRTALMGEQPNPWDRLQPQDAMSRHRGAKPPRRXXX

>exp_CellWallHydrolase_t14-104mBot_S017RuonanNODE_243_length_1179_cov_9.646780_g210_i1_1/1-152

------------------------------------------------------MSRHRGAKLPRRYEL

>exp_CellWallHydrolase_t14-104mBot_S017RuonanNODE_43785_length_377_cov_0.834646_g43658_i0_1/1-117

VRHGPLVLESAPLKFLTPATDRDRTVSRRSEPSSRAALMGEQLNPWDLLQPQDVTSRHRGAKPPRRCEL

>exp_CellWallHydrolase_t140mBot_S016RuonanNODE_91316_length_294_cov_2.345029_g91117_i0_1/1-77

VRLGPLVLESASLNVPTPTTDTDRTVSRRSEPSSRTTLTGEQPDPWDLLQP------------------

>exp_CellWallHydrolase_t14100mBot_S030RuonanNODE_106949_length_316_cov_1.751295_g105785_i0_1/1-100

------VLRSDPLKLPTITSDRDRTVSRRSEPSSRATLMSEQLNPWDLLQPQDVTSRHRGAKPPRRYGL

>exp_CellWallHydrolase_t1444mBot_S032RuonanNODE_221_length_1013_cov_120.666292_g114_i14_1/1-98

VRPGPLVLRTDLLKFPTRAADRDRTVSRRSEPSSRAALMGEQPNPWDRLQPQDAMSRHRGAKPPRRCGL

>exp_CellWallHydrolase_t1444mBot_S032RuonanNODE_8657_length_413_cov_0.000000_g8087_i0_1/1-42

--------------------------------------MGEQPNPWDLLQPQDVTSRHRGAKPFRRYEL

>exp_CellWallHydrolase_t1484mBot_S015RuonanNODE_29092_length_385_cov_2.988550_g28601_i0_1/1-72

VHPGPLVLRTAPLKFPTPATDRDRTVSRRSEPSSRAALMGEQPNPWDLLQPQDATSRHRG---------

>exp_CellWallHydrolase_t1484mBot_S015RuonanNODE_529_length_1363_cov_688.550806_g481_i5_1/1-144

VHPGPLVLRTAPLKFPAPATDRDRTVSRRSEPSSRTALMGEQPNPWDRLQPQDAMSRHRGAKPPRRCGL

>exp_CellWallHydrolase_t1492mBot_S04RuonanNODE_5377_length_456_cov_3.312312_g5118_i0_1/1-110

VHPGPLVLGTDPLKLRTPTADRDQTVSRRSEPSSRTTLIGEQPNPWDLLQPQDVMSRHRGAKLCRRYGL

>exp_CellWallHydrolase_t15104mBot_S031RuonanNODE_126356_length_280_cov_2.407643_g125738_i0_1/1-64

VRLGPLVLESGPLKSLTPITDRDRTVSRRSEPSSRATLIGEQPNPWDLLQPQ-----------------

>exp_CellWallHydrolase_t15104mBot_S031RuonanNODE_35804_length_411_cov_0.666667_g35470_i0_1/1-104

VHSGPLVLGAAPLKFPTPTMDRDRTVSRRSEPSSRTALIGERPNPWDVLPPQDATSRHRGAKQGRRCEL

>exp_CellWallHydrolase_t1532mBot_S023RuonanNODE_19348_length_403_cov_0.689286_g18125_i0_1/1-120

VHSGPLVLEAALLNYLTPTTDRDRTVSRRSEPNSRTALNGEQPYPWDLLQPQDAMSRHRGAKPLRRYEL

>exp_CellWallHydrolase_t1532mBot_S023RuonanNODE_22378_length_393_cov_3.618519_g21101_i0_1/1-67

------------------MVDRDRTVSRRSELSSRTALMGAQPNPWDLLQPQDATSRHRGAKLPRRCGL

>exp_CellWallHydrolase_t1532mBot_S023RuonanNODE_35318_length_357_cov_7.666667_g33226_i0_1/1-74

VRPGPLVLGTGFLKLLTRAADRDRTVSRRSKPSSRAALMGEQPNPWDL---------------------

>exp_CellWallHydrolase_t1532mBot_S023RuonanNODE_60725_length_308_cov_1.832432_g58577_i0_1/1-74

------------LNIPTPTVDRDRTVSRRSEPSSRTSLNGEQPYPWDLLQPQDEMSRHRGAKHHRRCGR

>exp_CellWallHydrolase_t1532mBot_S023RuonanNODE_9592_length_441_cov_2.880503_g8637_i0_1/1-123

VHPGPLVLRTAPLNTPTPTADRDRTVSRRSEPSSRTTLIGEQPNPWDLLQPQDVMSRHRGAKRSRRYGL

>exp_CellWallHydrolase_t1552mBot_S027RuonanNODE_121801_length_393_cov_4.825926_g115083_i0_1/13-130

IRPGPLVLGAGLRNSLSPTTDRDRTVSRRSEPSSRAALMGEQPNPWDLLRPQDATSRHRGAKPCRRCGL

>exp_CellWallHydrolase_t1552mBot_S027RuonanNODE_155703_length_378_cov_1.407843_g148789_i0_1/1-76

VHPGPLVLGTASLNSLTPTADRDRTVSRRSEPSSRTTLIGEQPNPWDLLQPQDVMSRHRGAKPPRRCGL

>exp_CellWallHydrolase_t1580mBot_S013RuonanNODE_135387_length_274_cov_2.218543_g134523_i0_1/1-91

VRPGPLVLRTAPLKFPSRTPDRDRTVSRRSEPSSRTALMGEQPNPSHLLQREDAMSRHRGAKPGRRCGL

>exp_CellWallHydrolase_t1580mBot_S013RuonanNODE_20_length_2950_cov_12.957198_g19_i1_1/1-152

VQHGPLVLVSDLRKFPAPTTDRDRTVSRRSEPSSRATLMGEQPNPWDLLQPQDVTSRHRGAKPFRRYEL

>exp_CellWallHydrolase_t1580mBot_S013RuonanNODE_25345_length_436_cov_3.421725_g24613_i0_1/1-104

VHPGPLVLRTDPLKYPAPAADRDRTVSRRSEPSSRTALIGEQPNPWNLLQLQDAMSRHRGAKPPRRCGL

>exp_CellWallHydrolase_t1580mBot_S013RuonanNODE_273_length_1459_cov_5.711826_g252_i1_1/1-152

VQRGPLVLVSAPLKSPTPTTDRDRTVSRRSEPSSRATLIGEQPNPWDLLQPQDVTSRHRGAKPPRRYEL

>exp_CellWallHydrolase_t1580mBot_S013RuonanNODE_51184_length_390_cov_0.958801_g50351_i0_1/1-122

VQFGPLVLESSPLKHLTPAIDKDQTVSRRSEPSSRATLMGEQPNPWDLLQPQDVTSRHRGAEPPRRCGL

>exp_CellWallHydrolase_t1592mBot_S010RuonanNODE_23774_length_406_cov_9.809187_g23321_i0_1/1-85

VFLGPLVLEKEPLKFPAPTVDKDRTVSRRSEPSSRAALTGEQPDPWDLLQPQDATRRHR----------

>exp_CellWallHydrolase_t1592mBot_S010RuonanNODE_29417_length_394_cov_0.601476_g28954_i0_1/1-41

--------------------------------------MGEQPNPWEVLPPQDATSRHRGAKPRRRCER

>exp_CellWallHydrolase_t1592mBot_S010RuonanNODE_5090_length_530_cov_2.255528_g4866_i1_1/45-172

VQPGPLVLGSGPRKFPAPTRDRDRTVSRRSEPSSRTALTGEQPDPWDLLQPQDAMSRHRGAKPGRRCEL

>exp_CellWallHydrolase_t16104mBot_S021RuonanNODE_5266_length_469_cov_2.300578_g5051_i0_1/1-66

------------------MADRDRTVSRRSEPSSRTALMGEQPNPWDLLQPQDATSRHRGAKPVRRCGL

>exp_CellWallHydrolase_t16104mBot_S021RuonanNODE_53083_length_292_cov_5.520710_g52709_i0_1/1-35

--------------------------------------MDEQPNPWDLLQPQDAMSRHRGAKPPRRCEL

>exp_CellWallHydrolase_t1632mBot_S012RuonanNODE_150319_length_312_cov_3.227513_g148407_i0_1/1-61

--------------AL----DMDRTVSRRSEPSSRTALMGEQPNPWDVLPPQVAMSRHRGAKPPRRCEL

>exp_CellWallHydrolase_t1632mBot_S012RuonanNODE_172309_length_300_cov_3.728814_g170390_i0_1/1-52

VQHGPLVLMSGPLKYPTPTTDRDRTVSRRSEPSSRATLMG-----------------------------

>exp_CellWallHydrolase_t1632mBot_S012RuonanNODE_207254_length_274_cov_8.781457_g205327_i0_1/1-76

---GPLVLGKGPLNALTPTPDMDRTVSRRSEPSSRTALMGEQPNPWNILQPQVAKSRHRGAKPSRRCKL

>exp_CellWallHydrolase_t1632mBot_S012RuonanNODE_227560_length_265_cov_2.380282_g225629_i0_1/1-58

VHPGPLVLGTDPVNIPTPTADRDRTVSRRSEPSSRTALIGEQPNPC-----------------------

>exp_CellWallHydrolase_t1632mBot_S012RuonanNODE_87099_length_375_cov_3.293651_g85236_i0_1/1-81

IPLGPLVLQRTSLKCPTRTTDRDRTVSRRSEPSSRTALMGEQPNPWEILLPQDAM--------------

>exp_CellWallHydrolase_t1644mBot_S01RuonanNODE_3905_length_532_cov_2.276284_g3720_i1_1/1-111

VRLGPLVLKTALLKIPPRAADRDRTVSRRSEPSSRAALMGEQPNPWDRLQPQDATSRHRGAKPCRRYER

>exp_CellWallHydrolase_t164mBot_S020RuonanNODE_17156_length_470_cov_0.703170_g16181_i0_1/1-154

------------------------------KPSSRTTLNGEQPYPWDLLQPRDVMSRHRGAKYRRRYEL

>exp_CellWallHydrolase_t164mBot_S020RuonanNODE_805_length_1037_cov_76.715536_g477_i7_2/1-218

VHSGPLVLGAAPFKFPTPTADRDQTVSRRFKPSSRTTLNGEQPYPWDRLQPQDVMSRHRGAKLPRRYEL

>exp_CellWallHydrolase_t164mBot_S020RuonanNODE_9643_length_519_cov_5.378788_g8726_i1_1/1-136

VHPGPLVLGTNPLNSPTPTTDRDRTVSRRSEPSSRTTLIGEQPNPWDLLQPQDVISRHRGAKRCRRYGL

>exp_CellWallHydrolase_t1660mBot_S022RuonanNODE_1135_length_693_cov_4.978947_g1056_i1_1/1-132

VQPGPLVLRLEPLKFPAPTVDKDRTVSRRSEPSSRTALTGEQPDPWDLLQPQDAMSRHRGAKPSRRYEL

>exp_CellWallHydrolase_t1660mBot_S022RuonanNODE_190_length_1122_cov_2117.888889_g160_i1_1/1-118

CVSGPLVLGAAPLSSPAPTADRDRTVSRRSKPSSRTTLNGEQPYPWDLLQPQDVMSRHRGAKHRRRYEL

>exp_CellWallHydrolase_t1660mBot_S022RuonanNODE_607_length_801_cov_12.675516_g543_i2_1/1-145

VQRGPLVLVSAPLKSPTPTTDRDRTVSRRSEPSSRATLIGEQPNPWDLLQPQDVTSRHQGAKPLRRYEL

>exp_CellWallHydrolase_t1684mBot_S019RuonanNODE_25809_length_367_cov_6.450820_g25436_i0_1/1-91

-----LVLRKAPLKYRAPTVDRDRTVSRRSEPSSRAALIGEQPNPWDLLQPQDATSRHRGAKPRRRCER

>exp_CellWallHydrolase_t1692mBot_S026RuonanNODE_50039_length_319_cov_10.535714_g49045_i0_1/1-77

VHPGPLVLRTGLLKSHTPTEDKDQTVSRRSEPSSRTTLISEQLNPWDLLQP------------------

>exp_CellWallHydrolase_t1720mBot_S033RuonanNODE_123942_length_364_cov_2.186722_g121885_i0_1/1-91

VHPGPLVLGTAPLKYPAPTTDRDRTVSRRSEPSSRTALIGEQPNPWDLLQPQDAMSRHRGAKPRR----

>exp_CellWallHydrolase_t1732mBot_S06RuonanNODE_45904_length_349_cov_3.486726_g45318_i0_1/1-105

VILGPLVLENEPLKFPAPTVDKDRTVSRRSEPSSRTALTGEQPDPWDLLQPQDAMSRHRGAKPVRRYEL

>exp_CellWallHydrolase_t1732mBot_S06RuonanNODE_80959_length_286_cov_2.098160_g80355_i0_1/1-72

VRLGPLVLESGPLKLQTPTTDRDRTVSRRSEPSSRATLIGEQPNPW-----------------------

>exp_CellWallHydrolase_t174mBot_S011RuonanNODE_3750_length_494_cov_1.574124_g3619_i1_1/1-78

IRPGPLVLGSAPLNLPTPTADTDRTVSRRSEPSSRTTLTGEQPDPWDLLQPQ-----------------

>exp_CellWallHydrolase_t1760mBot_S028RuonanNODE_16071_length_458_cov_13.835821_g14966_i0_1/23-139

VHPGPLVLRTAPPKFPAPATDRDRTVSRRSEPSSHTALMGEQPNPWDRLQPQDEMSRHRGAKPPRRCGL

>exp_CellWallHydrolase_t1764mBot_S05RuonanNODE_69111_length_347_cov_2.151786_g68341_i0_1/1-115

VRLGPLVLESGPFKYHTPTTDRDRTVSRRSEPSSRAALMGEQPNPWDLLQPQDVTSRHRGAKPPRRYEL
